# Trends of Axillary Treatment in Sentinel Node-Positive Breast Cancer Patients Undergoing Mastectomy

**DOI:** 10.1245/s10434-023-13568-3

**Published:** 2023-05-24

**Authors:** Eline E. F. Verreck, Julia E. C. van Steenhoven, Anne Kuijer, Marissa C. van Maaren, Janine M. Simons, Sabine Siesling, Thijs van Dalen

**Affiliations:** 1 University Utrecht, Utrecht, The Netherlands; 2 Department of Surgery, Diakonessenhuis Utrecht, Utrecht, The Netherlands; 3 Department of Pathology, University Medical Centre Utrecht, Utrecht, The Netherlands; 4 Department of Surgery, St. Antonius Hospital, Nieuwegein, The Netherlands; 5 Department of Research and Development, Netherlands Comprehensive Cancer Organisation (IKNL), Utrecht, The Netherlands; 6 Department of Health Technology and Services Research, Technical Medical Centre, University of Twente, Enschede, The Netherlands; 7 Department of Radiotherapy, Erasmus MC, Rotterdam, The Netherlands; 8 GROW school for oncology and reproduction, MUMC+, Maastricht, The Netherlands; 9 Department of Surgery, Erasmus MC, Rotterdam, The Netherlands; 10 Department of Surgery, University Medical Centre Utrecht, Utrecht, The Netherlands

## Abstract

**Background:**

The ACOSOG-Z0011- and the AMAROS-trial obviated the need for axillary surgery in most sentinel node-positive (SLN+) breast cancer patients undergoing breast-conserving surgery (BCS). Data for patients who undergo mastectomy is scarce. The purpose of this study was to investigate patterns of axillary treatment in SLN+ patients treated by mastectomy in the years after the publication of landmark studies regarding axillary treatment in SLN+ breast cancer patients undergoing BCS.

**Methods:**

This was a population-based study in cT1-3N0M0 breast cancer patients treated by mastectomy and staged as SLN+ between 2009 and 2018. The performance of an axillary lymph node dissection (ALND) and/or administration of postmastectomy radiotherapy (PMRT) were primary outcomes and were studied over time.

**Results:**

The study included 10,633 patients. The frequency of ALND performance decreased from 78% in 2009 to 10% in 2018, whereas PMRT increased from 4 to 49% (*P* < 0.001). In ≥N1a patients, ALND performance decreased from 93 to 20%, whereas PMRT increased to 70% (*P* < 0.001). In N1mi and N0itc patients, ALND was abandoned during the study period, whereas PMRT increased to 38% and 13% respectively (*P* < 0.001), respectively. Age, tumor subtype, N-stage, and hospital type affected the likelihood that patients underwent ALND.

**Conclusions:**

In this study in SLN+ breast cancer patients undergoing mastectomy, use of ALND decreased drastically over time. By the end of 2018 most ≥N1a patients received PMRT as the only adjuvant axillary treatment, whereas the majority of N1mi and N0itc patients received no additional treatment.

During the past decade, several randomized trials have cast doubt on the need to perform axillary lymph node dissection (ALND) in patients with sentinel lymph node metastases (SLN+). The Z0011 trial of The American College of Surgeons Oncology Group (ACOSOG), published in 2011, demonstrated that ALND in cT1-2 patients undergoing breast-conserving surgery (BCS) who were found to have one or two positive SLN (SLN+) showed no lower regional recurrence risk or better survival compared with those undergoing sentinel lymph node biopsy (SLNB) only.^1,2^ The International Breast Cancer Study Group trial (IBCSG 23-01) showed similar results for patients with micrometastases in the SLN.^3^ The results of the “After Mapping of the Axilla: Radiotherapy or Surgery?” (AMAROS) trial, published in 2014, demonstrated that axillary radiotherapy (RT) could serve as a safe alternative to ALND resulting in equivalent regional control.^4^

The results of these trials led to a broad discussion about the need of performing ALND in SLN+ patients and about the use of RT as an alternative to ALND in SLN+ patients who would previously had been candidates for ALND. International guidelines suggest to consider foregoing axillary surgery in patients meeting the Z0011 criteria, i.e., patients who were treated by breast-conserving surgery (BCS) followed by routine external beam RT of the breast.^5–7^ Other guidelines advocate the use of regional RT as an alternative for ALND in SLN+ patients,^6^ applying the AMAROS results both to patients who undergo BCS as well as to patients treated by mastectomy.

Some years ago, a substantial decrease was reported in ALND frequency among SLN+ patients both in those undergoing BCS and mastectomy.^8,9^ In a previous Dutch population-based study, describing patients treated from 2011 to 2015, the proportion of SLN+ patients receiving ALND alongside BCS versus mastectomy was 31% versus 52% at the start but had decreased to 11% and 26%, respectively, by the end of the study period.^8^ These trend lines show a stronger reduction of ALND in the context of BCS versus mastectomy, which may reflect an altered protocol with regard to the anticipated effectivity of ALND in conjunction with BCS. Because for mastectomy patients the Z0011 criteria do not apply, one might expect that postmastectomy radiotherapy (PMRT) would have been applied as a substitute for ALND.

Therefore, the purpose of this study was to investigate patterns of care in axillary treatment for Dutch cT1-3N0 SLN+ breast cancer patients undergoing mastectomy. Furthermore, patient-, tumor-, treatment-, and hospital-related factors that are associated with ALND performance were evaluated.

## Methods

Data were obtained from the nationwide population-based Netherlands Cancer Registry (NCR), which is hosted by the Netherlands Comprehensive Cancer Organisation (IKNL). Based on notification through the national pathology database (PALGA) specially trained IKNL data managers register patient-, tumor-, and treatment-related characteristics directly from the patient’s files.

### Patients and Hospitals

For the present study, all Dutch adult female patients diagnosed with cT1-3N0M0 invasive breast cancer who underwent mastectomy including SLNB between January 2009 and December 2018 were selected from the NCR. Patients who had SLNs containing metastases were included. Those who received neoadjuvant systemic therapy, underwent mastectomy without SLN biopsy, as well as patients in whom the SLN could not be identified intraoperatively were excluded.

### Construction of Variables

Patients were subdivided in groups according to axillary treatment following SLNB: ALND, PMRT, a combination of the two (ALND + PMRT), or no subsequent axillary treatment. Detailed information regarding radiation fields was not available. In the Netherlands, the indication for RT of the chest wall in the primary setting is dependent on the estimated risk of recurrence and the absence or presence of risk factors. In case regional RT is indicated in postmastectomy patients (dependent on the extent of nodal disease and the absence or presence of risk factors), the chest wall is generally included in the radiotherapy field. Metastatic lymph node involvement was categorized into isolated tumor cells (N0itc), micrometastases (N1mi) or macrometastases (≥N1a) based on the pathology examination of the retrieved SLNs. Hospitals were categorized based on surgical hospital volume. They were divided into low volume (<150 breast cancer operations for primary breast cancer), middle volume (150-300 operations), and high volume (>300 operations) on average per year. Cutoff points were based on those reported by EUSOMA, the European Society of Breast Cancer Specialists,^10^ and those reported in an article from Greenup et al.^11^ Hospitals also were categorized by their teaching status as general nonteaching, teaching, or academic centers.

### Statistical Analysis

Patient-, tumor-, treatment-, and hospital-related characteristics are presented as baseline characteristics according to the different treatment groups and compared by using chi-squared tests. Descriptive analyses were used to report on the annual proportions of axillary treatments. Univariable and multivariable logistic regression analyses were used to identify patient-, tumor-, treatment-, and hospital-related factors that are associated with ALND performance. *P* value < 0.05 was considered statistically significant. Data analyses were performed using Stata version 17.0 (StataCorp, TX).

## Results

### Patients

In total 10,633 patients were included in the analysis. Most of the SLN+ patients were diagnosed with a cT1-2 tumor (93%, *n* = 9864). The remaining 7% of the patients were diagnosed with a cT3 tumor (*n* = 769; Table 1). In most of the patients receiving SLNB alone and no ALND (*n* = 6457), one to three lymph nodes were removed and examined (83%, *n* = 5355; median 2; IQR 1-3).

**Table 1:** Baseline characteristics of all SLN+ patients treated with ALND, ALND + PMRT, PMRT, or no adjuvant axillary treatment (*n* = 10,633)

Characteristics	*N* overall	ALND		ALND + PMRT		PMRT		No adjuvant axillary treatment	
	*N*	*N*	%	*N*	%	*N*	%	*N*	%
Year of diagnosis									
2009	854	462	54	206	24	33	4	153	18
2010	1041	561	54	198	19	52	5	230	22
2011	1205	537	45	216	18	121	10	331	28
2012	1197	431	36	187	16	180	15	399	33
2013	1157	342	30	134	12	258	22	423	37
2014	1142	205	18	132	12	362	32	443	39
2015	1098	137	13	75	7	477	43	409	37
2016	1008	86	9	56	6	458	45	408	41
2017	982	67	7	49	5	451	46	415	42
2018	949	64	7	31	3	463	49	391	41
Age group (year)									
< 40	556	165	30	111	20	150	27	130	23
40–49	1979	624	32	317	16	492	25	546	28
50–59	2521	774	31	328	13	681	27	738	29
60–69	2498	675	27	311	13	681	27	831	33
70–79	1754	426	24	150	9	547	31	631	36
> 79	1325	228	17	67	5	304	23	726	55
Histological tumour type									
Ductal	7372	2193	30	836	11	1850	25	2493	34
Lobular	2419	493	20	361	15	750	31	815	34
Mixed	614	152	25	65	11	208	34	189	31
Other	228	54	24	22	10	47	21	105	46
Differentiation grade									
I	1806	544	30	159	9	419	23	684	38
II	5808	1502	26	684	12	1557	27	2065	36
III	2759	755	27	413	15	833	30	758	28
Unknown	260	91	35	28	11	46	18	95	37
Clinical tumour stage									
cT1	4730	1466	31	444	9	1054	22	1766	37
cT2	5134	1330	26	694	14	1453	28	1657	32
cT3	769	96	13	146	19	348	45	179	23
Multifocality									
No	7165	2002	28	867	12	1739	24	2557	36
Yes	3424	877	26	408	12	1107	32	1032	30
Unknown	35	7	20	9	26	7	20	12	34
Breast cancer subtype									
HR+/HER2−	8535	2290	27	999	12	2345	28	2901	34
HR+/HER2+	899	260	29	108	12	218	24	313	35
HR−/HER2+	359	108	30	57	16	82	23	112	31
HR−/HER2−	625	193	31	104	17	156	25	172	28
Other/unknown	215	41	19	16	7	54	25	104	48
Pathological N-stage									
Isolated tumour cells	2170	70	4	17	1	224	10	1850	85
Micrometastasis	2847	743	26	104	4	739	26	1261	44
Macrometastasis	5616	2070	37	1163	21	1892	34	491	9
Hormonal therapy									
No	2106	509	24	234	11	461	22	902	43
Yes	8527	2383	28	1050	12	2394	28	2700	32
Chemotherapy									
No	5199	1101	21	243	5	1360	26	2495	48
Yes	5434	1791	33	1041	19	1495	28	1107	20
Hospital volume									
< 150 resections per year	4277	1277	30	512	12	1021	24	1467	34
150–300 resections per year	5951	1515	26	733	12	1685	28	2018	34
> 300 resections per year	400	99	25	37	9	148	37	116	29
Hospital type									
General nonteaching	4604	1288	23	498	11	1247	27	1571	34
Teaching hospital	5130	1402	27	707	14	1334	26	1687	33
Academic hospital	894	201	23	77	9	273	31	343	38

*ALND* axillary lymph node dissection, *PMRT* postmastectomy radiotherapy, *HR* hormone receptor, *HER2+* human epidermal growth factor receptor 2, *SLNB* sentinel lymph node biopsy

### Trends in Axillary Treatment in cT1-3 SLN+ Breast Cancer Patients Undergoing Mastectomy

**Fig. 1:**
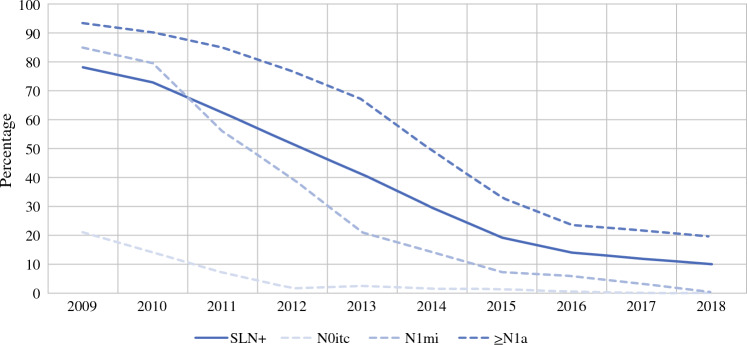
Frequency of ALND in sentinel node-positive patients undergoing amputation. *SLN+* sentinel lymph node positive, *N0itc* isolated tumor cells, *N1mi* micrometastases, ≥*N1a* macrometastases

The proportion of SLN+ patients who underwent ALND following mastectomy (*n* = 10,633) decreased from 78% in 2009 to 10% in 2018 (Fig. 1). The frequency of ALND decreased from 93 to 20% in ≥N1a patients, from 85% to 0.4% in N1mi patients, and from 21% to 0% in N0itc patients, respectively (Fig. 1).

**Fig. 2:**
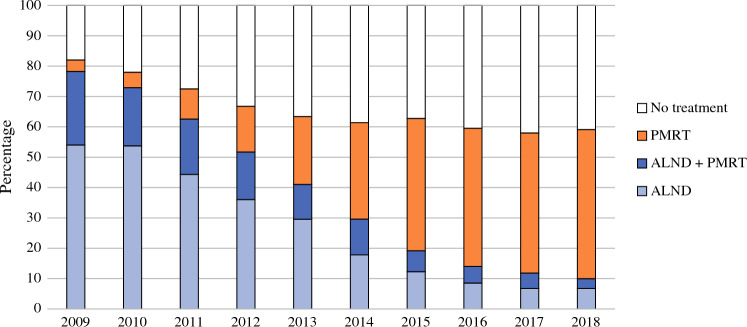
Frequency of axillary treatment in sentinel node-positive patients. *PMRT* postmastectomy radiotherapy, *ALND* axillary lymph node dissection

Figure 2 shows the trend of adjuvant axillary treatment. Both ALND and ALND combined with PMRT decreased from 54% in 2009 to 7% in 2018 and from 24 to 3%, respectively. The use of PMRT as the only type of adjuvant treatment increased from 4 to 49% (*P* < 0.001 for all). For patients with a cT3 tumor, ALND (ALND alone or combined with PMRT) decreased from 72 to 13%. Excluding patients with T3 tumors had no significant impact on the results for the whole group. In the selection of patients with cT1-2 tumors, the proportion of ALND decreased from 55 to 7% and treatment with PMRT increased from 4 to 48%.

**Fig. 3:**
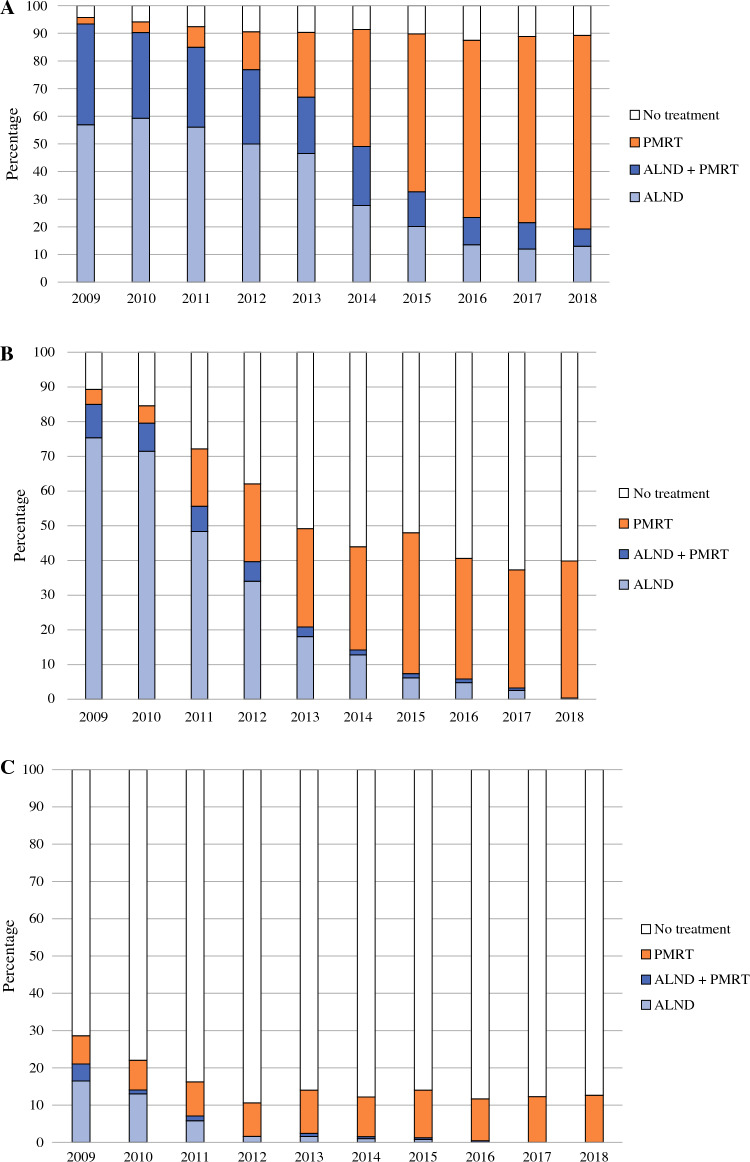
**A** Frequency of axillary treatment in ≥N1a patients. **B** Frequency of axillary treatment in N1mi patients. **C** Frequency of axillary treatment in N0itc patients. *PMRT* postmastectomy radiotherapy, *ALND* axillary lymph node dissection

The trends of adjuvant axillary treatment varied for the different N+ categories groups. In ≥N1a patients, the increase of PMRT from 2% in 2009 to 70% in 2018 was accompanied by a decrease in ALND from 57 to 13% (*P* < 0.001 for all; Fig. 3a). In the N1mi group, the decrease of ALND appeared most prominent from 75 to 0.4% (*P* < 0.001; Fig. 3b). This decrease in ALND performance was only in part accompanied by an increase of PMRT from 4 to 38% (*P* < 0.001). In the latter years, a substantial number of patients did not receive axillary treatment at all. In N0itc patients, ALND was abandoned rapidly from 17% to approximately 0% since 2012 (*P* < 0.001; Fig. 3c). The use of PMRT being approximately 10% throughout the study period.

### Patients-, Tumor-, and Hospital Characteristics which Influence the Choice of Omitting ALND

In addition to the effect of time, factors that were associated with a decreased chance of undergoing ALND were patients > 79 years (odds ratio [OR] 0.27; 95% confidence interval [CI] 0.21-0.35) compared with age 50-59 years, treatment with PMRT (OR 0.14; 95% CI 0.12–0.17), patients with tumor’s differentiation grade II (OR 0.83; 95% CI 0.70–0.98) compared with grade I, and patients with sentinel nodes containing isolated tumor cells (OR 0.00; 95% CI 0.00–0.01) or micrometastases (OR 0.10; 95% CI 0.08–0.11) compared with macrometastases.

Factors that were associated with a higher chance of ALND performance were age < 40 years (OR 1.28; 95% CI 0.96–1.70) compared with age 50-59 years, lobular (OR 1.23; 95% CI 1.05–1.43) compared with ductal tumor type, basal-like (OR 1.83; 95% CI 1.33–2.53) compared with hormone receptor-positive (HR+)/HER2 receptor-negative tumor subtype, receiving chemotherapy (OR 2.34; 95% CI 1.98–2.77) compared with not receiving adjuvant chemotherapy, as well as treatment outside an academic institution (teaching hospital: OR 2.19; 95% CI 1.71–2.81, general hospital: OR 1.58; 95% CI 1.25–2.00) (Table 2).

**Table 2:** Univariable and multivariable analysis patient, tumor, and hospital characteristics associated with the performance of ALND

	Univariable				Multivaria﻿ble	
	*N*	% ALND	Odds ratio	95% CI	Odds ratio	95% CI
Year of incidence						
2009	854	78	1.334	1.079–1.650	1.65	1.20–2.27
2010	1041	73	Ref		Ref	
2011	1205	62	0.619	0.517–0.741	0.45	0.34–0.59
2012	1197	52	0.397	0.332–0.474	0.21	0.17–0.28
2013	1157	41	0.260	0.217–0.311	0.12	0.09–0.16
2014	1142	30	0.156	0.129–0.187	0.08	0.06–0.10
2015	1098	19	0.089	0.073–0.109	0.04	0.03–0.05
2016	1008	14	0.061	0.049–0.076	0.03	0.02–0.04
2017	982	12	0.050	0.039–0.063	0.02	0.02–0.03
2018	949	10	0.041	0.032–0.053	0.02	0.01–0.03
Age (year)						
< 40	556	50	1.27	1.06–1.53	1.32	1.00–1.76
40–49	1.979	48	1.17	1.04–1.31	1.08	0.86–1.25
50–59	2521	44	Ref		Ref	
60–69	2498	39	0.84	0.75–0.94	1.04	0.88–1.24
70–79	1754	33	0.63	0.55–0.71	0.93	0.74–1.16
> 79	1325	22	0.37	0.32–0.43	0.27	0.21–0.35
Histological tumor type						
Ductal	7372	41	Ref		Ref	
Lobular	2419	35	0.78	0.71–0.86	1.23	1.05–1.43
Mixed	614	35	0.78	0.66–0.93	0.86	0.66–1.12
Other	228	33	0.72	0.54–0.95	0.91	0.59–1.42
Differentiation grade						
I	1806	39	Ref		Ref	
II	5808	38	0.95	0.85–1.06	0.83	0.70–0.98
III	2759	42	1.15	1.02–1.30	1.00	0.82–1.22
Unknown	260	46				
Clinical tumour stage						
cT1	4730	40	Ref		Ref	
cT2	5134	39	0.96	0.89–1.04	1.08	0.95–1.23
cT3	769	31	0.68	0.58–0.80	1.11	0.87–1.41
Multifocality						
No	7165	40	Ref		Ref	
Yes	3424	38	0.90	0.83–0.98	0.98	0.86–1.11
Unknown	35	46				
Pathological N-stage						
Isolated tumour cells	2170	4	0.034	0.028–0.042	0.00	0.00–0.01
Micrometastasis	2847	30	0.312	0.284–0.344	0.10	0.08–0.11
Macrometastasis	5616	58	Ref		Ref	
Breast cancer subtype						
HR+/HER2−	8535	39	Ref		Ref	
HR+/HER2+	899	41	1.11	0.96–1.27	0.75	0.60–0.93
HR−/HER2+	359	46	1.36	1.10–1.68	1.15	0.78–1.69
HR−/HER2−	625	48	1.44	1.23–1.70	1.83	1.33–2.53
Unknown	215	27	0.58	0.42–0.78	0.72	0.45–1.16
Adjuvant hormonal therapy						
No	2106	35	Ref		Ref	
Yes	8527	40	1.24	1.12.–1.37	1.26	1.02–1.55
Adjuvant chemotherapy						
No	5199	26	Ref		Ref	
Yes	5434	52	3.12	2.88–3.39	2.34	1.98–2.77
Radiotherapy						
No	6494	45	Ref		Ref	
Yes	4139	31	0.56	0.52–0.61	0.14	0.12–0.17
Hospital volume						
Low (< 150)	4277	42	Ref		Ref	
Medium (150–300)	5951	38	0.84	0.78–0.91	0.75	0.65–0.86
High (> 300)	400	34	0.72	0.58–0.89	0.91	0.65–1.26
Hospital type						
Academic	894	31	Ref		Ref	
Teaching	5130	41	1.55	1.33–1.80	2.19	1.71–2.81
General	4604	39	1.40	1.20–1.64	1.58	1.25–2.00

*ALND* axillary lymph node dissection (with or without postmastectomy radiotherapy); *HR* hormone receptor; *HER2+* human epidermal growth factor receptor 2; *SLNB* sentinel lymph node biopsy

## Discussion

In this population-based study in Dutch cT1-3N0M0 breast cancer patients who underwent mastectomy and were SLN+, a substantial decrease in the proportion of patients undergoing ALND was observed. In patients diagnosed with ≥N1a disease, ALND performance decreased and PMRT increased substantially over the years, whereas in patients with isolated tumor cells and micrometastasis, a substantial proportion had no adjuvant regional treatment at the end of the study period.

Ten years after the publication of the Z0011 and AMAROS trials, the proportion of Dutch patients undergoing mastectomy who were SLN+ and underwent ALND decreased to 10%. This seems to reflect the clinicians’ confidence in a restrained surgical policy in this category of patients, albeit that the aforementioned trials included patients undergoing BCS exclusively (Z0011) or mostly (82% in the AMAROS trial).^2,4,12^ A recent population-based study from the United States in a similar cohort of 12,190 patients also showed a decrease in the proportion patients undergoing ALND from 58% in 2005 to 36% in 2014,^13^ whereas another large, population-based study in Germany showed a decrease from 90% in 2008 to 56% in 2015.^14^

The present study shows replacement of ALND with PMRT as axillary treatment after mastectomy in patients staged as ≥N1a. While only 20% of ≥N1a patients underwent ALND at the end of the study period, 70% received PMRT. This trend to omit ALND and increasingly use PMRT has been reported by others,^13,15^ arguing in favor of this treatment switch citing the evidence from the AMAROS trial results. In addition, a remarkable decrease in both performing ALND and administering PMRT as adjuvant axillary treatment is observed. Others also reported this decrease.^16^ Proceeding with PMRT instead of ALND in SLN+ patients precludes the identification of patients with N2 or N3 disease. Long-term outcome remains to be awaited, but the short-term advantage in terms of less arm morbidity when fewer patients undergo both local treatment modalities goes without saying.

In N0itc and N1mi patients, the decreasing trend in axillary surgery was observed earlier during the study period and the decrease was to a lesser extent accompanied by an increase in PMRT compared with ≥N1a patients. This may partly be clarified by the Dutch breast cancer treatment guideline from 2012,^17^ which recommended that adjuvant axillary treatment was unnecessary in N0itc patients and questioned the need of axillary treatment in a selection of N1mi patients, e.g., depending on the number of lymph nodes that contained micrometastasis or the presence of other risk factors, such as young age (<40 years), grade 3 disease, lymphovascular invasion, or triple-negative disease. The conceivable association between the degree of metastatic lymph node involvement and the proportion of patients who undergo axillary surgery also was observed by others.^13–15,18^ Apart from the observed decreased performance of ALND, the association between the extent of metastatic involvement of the SLN and the subsequent administration of PMRT suggests that in SLN+ patients who undergo mastectomy and are diagnosed with ≥N1a disease, the AMAROS trials results are adhered to, whereas in patients with N1mi and N0itc, adjuvant treatment is considered unnecessary by many clinicians in the majority of patients.^3,16,19^

In addition to N-stage and histologic subtype of the tumor, several other factors were associated with the decision of whether to perform ALND. Women older than age 79 years had a lower chance of undergoing ALND, whereas women younger than age 40 years and women with basal-like tumor subtype had a higher chance of undergoing ALND.^14,15^ It seems that surgeons are more reserved in omitting ALND in young patients with an aggressive tumor subtype, albeit that a recent study suggests that clinicians may forego ALND in young patients when PMRT will be administered.^20^ Furthermore, the results of our study showed that patients who undergo adjuvant chemotherapy also were more likely to receive ALND. The higher likelihood of macrometastatic disease or high-grade disease in patients undergoing adjuvant chemotherapy probably contributes to this correlation, albeit that hospital type and the innovative characteric within a hospital also influences the use of systemic therapies and axillary treatment.

Albeit that patients with a cT3 tumor were not included in the Z0011 and AMAROS trial, we decided to include these patients in our dataset to evaluate patterns of care for this particular subgroup. Despite the lack of evidence to deescalate axillary treatment within this category of patients, the results of our study illustrated a similar decreasing trend in the performance of ALND in patients with cT3 tumors compared with those with T1-2 tumors.

The finding that patients treated outside an academic hospital were more likely to undergo axillary surgery is in line with the findings of another study from the Netherlands^8^ but contrasts with the opposite finding of three cohort studies from the United States and Germany.^13–15^ In the German study, patients who were treated in community cancer centers, in comparison to academic cancer centers, were more likely to undergo treatment with SLN dissection without ALND or PMRT (37.4% and 32.1%, respectively).^14^ Weiss et al. showed similar results; 37-38% of the patients treated in community centers only underwent SLN biopsy versus 32% in academic centers. The latter authors also observed that patients with public insurance were more likely to receive SLN biopsy only.^13^ Then again, in another American study, it was observed that patients undergoing an upfront ALND were more likely to be treated in a community center than those undergoing SLN biopsy alone.^15^ This all implies that opinions regarding axillary treatment differ between institutions, clinicians, and surgical societies.

The main strengths of the present study are the size of the study population, the quality of the items that were uniformly registered by personnel of the NCR, and the study period of 10 years. As a result, robust data regarding treatment trends are presented. Some limitations of the study are the absence of the number of removed and examined sentinel nodes in patients who underwent ALND following SLN biopsy, the timing of axillary surgery (SLN biopsy with the ALND versus delayed ALND), and the absence of information regarding the radiation fields. In the Netherlands, the indication for RT of the chest wall in the primary setting is dependent on the estimated risk of recurrence and the absence or presence of risk factors. If case regional RT is indicated in postmastectomy patients (dependent on the extent of nodal disease and the absence or presence of risk factors), the chest wall is generally included in the radiotherapy field. Another important limitation of the study design is the absence of follow-up information, because this is not routinely collected for all patients in the NCR. While evidence from clinical trials support the interchangeability of ALND and regional RT in patients treated with BCS, we are still awaiting the results of several clinical trials exploring the impact of omitting adjuvant local treatment in SLN+ patients who undergo mastectomy.^21–23^ These trials mostly included patients treated with BCS, whereas data specifically for patients undergoing mastectomy is scarce. The Dutch BOOG 2013-07 registry study assessed the oncologic safety of different extents of additional axillary treatment following a positive SLN, specifically in patients who underwent mastectomy.^24^ Follow-up of this trial was recently completed. While awaiting the results of these trials to determine optimal axillary treatment strategies in postmastectomy patients with a positive SLN, it seems sensible to avoid treating patients with both ALND and regional RT, because this combination is associated with the worst patient-reported outcomes compared with less invasive axillary treatments (SLNB or regional RT only).^25^ Based on the results of our study, specialists seem to already actively avoid this combination in daily practice, because these rates decreased further each year.

## Conclusions

This study shows a descending trend in the execution of ALND in SLN+ Dutch cT1-3N0M0 breast cancer patients undergoing mastectomy within the 10 years following the AMAROS and Z0011 trial results. ALND was omitted in the vast majority of SLN+ patients. In ≥N1a patients, PMRT increased drastically, whereas less than half of N1mi and only a tenth of N0itc patients received PMRT as the only adjuvant axillary treatment by the end of 2018.

## Data Availability

The data that support the findings of this study are available upon reasonable request.
